# Effects of *Dendrobium nobile* on antioxidant capacity, hormone levels, testicular metabolism, and reproductive performance of aged roosters

**DOI:** 10.1371/journal.pone.0322853

**Published:** 2025-05-09

**Authors:** Depeng Zhao, Yushi Shi, Xia Long, Qisong Tan, Jinlin Yang, Hui Li

**Affiliations:** 1 Key Laboratory of Animal Genetics, Breeding and Reproduction in the PlateauMountainous Region, Ministry of Education, Guiyang, Guizhou Province, China; 2 Guizhou Provincial Key Laboratory of Animal Genetic Breeding and Breeding, Guiyang, Guizhou Province, China; 3 College of Animal Science, Guizhou University, Guiyang, Guizhou Province, China; University of Hyderabad, INDIA

## Abstract

Oxidative stress is a major cause of semen quality decline in old roosters. *Dendrobium nobile* Lindl (DNL), a Chinese herbal medicine, exhibits excellent antioxidant activity. Therefore, the present study aimed to investigate the effects of dietary DNL supplementation on semen quality, antioxidant capacity, reproductive hormone levels, and testicular tissue structure in aged roosters. This study further aimed to elucidate the potential mechanism for improving reproductive performance. Thus, the expression of antioxidant defense system-related genes was verified, and metabolomic analysis was performed. Twenty 56-week-old recessive, white-feathered roosters were randomly assigned into two groups. The DNL group was fed a basal diet supplemented with 2500 mg/kg DNL for 60 days, whereas the control group was fed a basal diet. Here, DNL improved the semen quality (sperm density and motility) and antioxidant capacity of aged roosters, increased the expression of genes in the Nrf2 pathway, increased serum hormone levels, and delayed testicular tissue degradation. Seventy-six differential metabolites that are mainly enriched in amino acid biosynthesis pathways, taurine and hypotaurine metabolism, and cysteine and methionine metabolism were identified. DL-serine, DL-cysteine, and α-ketoglutarate were related to improved testicular antioxidant capacity. In this study, dietary supplementation with 2500 mg/kg DNL delayed the decline in reproductive performance by improving the antioxidant capacity of aging roosters. These findings could facilitate the use of DNL as a feed additive to improve the reproductive performance of aged roosters.

## Introduction

The semen quality of roosters directly affects fertilization, hatching, and healthy chick rates in poultry production, which has an economic impact on farms [1]. However, the reproductive performance of roosters is considerably affected by their age. Sperm collection, motility, and density, as well as hormone levels decrease after 50 weeks of age [2,3]. Reactive oxygen species (ROS), which are related to weakened antioxidant defense capabilities, increase with tissue aging [4]. The ROS production rate is substantially high in the testes, owing to the large amounts of oxygen required to produce steroids and sperm [5]. However, low ROS levels are necessary for normal sperm function and fertilization in the male reproductive system. High ROS levels can lead to excessive oxidative stress (OS), affecting all aspects of the reproductive system and semen, including sperm concentration, motility, and morphology [6,7]. Therefore, improved antioxidant capacity may alleviate the age-related decline in the reproductive performance of roosters.

Dietary supplementation with natural antioxidants, such as linseed oil [8], natural astaxanthin [9], and curcumin [10], can reduce age-related decline in semen quality. Delaying the decline in reproductive performance of roosters caused by aging can improve the utilization rate of excellent roosters and reduce the breeding cost of hatching egg farms and local chicken breed protection. *Dendrobium nobile* Lindl (DNL), a traditional Chinese medicine, is rich in alkaloids, polysaccharides, phenanthrenes, fluorenones, and phenylpropanoids. It has excellent anti-aging, anti-oxidant, hypoglycemic, antitumor, and immune-enhancing effects [11]. *Dendrobium* polysaccharides can reverse the oxidative damage caused by cyclophosphamide to the reproductive system of male mice [12]. In addition, they can improve testicular damage and spermatogenic disorders in pathological mice [13,14]. However, only a few studies have investigated the reproductive performance of aging roosters following DNL supplementation.

This study hypothesized that DNL can alleviate the age-related decline in reproductive performance by enhancing the antioxidant capacity of roosters. Therefore, DNL was added to the diet of aged roosters to explore its effects on the semen quality, hatching rate, testicular tissue structure, antioxidant indices, and reproductive hormone levels of roosters. This study further sought to explore the potential mechanism affecting reproductive performance by measuring the expression of antioxidant-related genes and performing metabolomic analysis.

## Materials and methods

### Ethical statement

All methods were performed in accordance with the relevant guidelines and regulations provided by the Regulations of the Administration of Affairs Concerning Experimental Animals (China, 1998) for animal experiments. All animal procedures were approved by the Animal Ethics Committee of Guizhou University (Approval No. EAE-GZU-2023-T096). This study was carried out in strict accordance with the recommendations in the Guide for the Care and Use of Laboratory Animals of the National Institutes of Health.

### Experimental animals and feeding experiments

Twenty 56-week-old recessive, white-feathered roosters (at the late breeding stage, the reproductive performance was in the recession stage [15], adapt to semen collection, and the body weight was 2.5 ± 0.2 kg) were selected from the scientific research chicken farm of Guizhou University. All roosters were selected from the same batch in the same house, and each rooster was reared in a single cage. The environment before and after feeding did not change, and the roosters were pre-fed for seven days before the experiment to avoid stress. The roosters were randomly divided into two groups and one bird per cage (n = 10 per group). The formal feeding period was 60 d. The control group was fed a basal diet, and the DNL group was fed a basal diet supplemented with DNL 2500 mg/kg. The DNL was offered by Guizhou Chishui Dendrobium nobile Industry Development Co., Ltd. (Chishui, Guizhou, China). After drying and crushing, DNL was mixed with the basal diet and then fed. The DNL supplementation was optimized based on previous studies [16]. All experimental roosters were under the same conditions: light 16 h/d, temperature 21 °C, humidity 45–65%, ad libitum access to water and diets. The feed composition and the main components of DNL were listed in Table 1 and Table 2.

**Table 1:** Ingredients for rooster feed.

Ingredients	Content (%)
Corn	74
Corn germ meal	5
Soybean meal (46%)	12
Soybean oil	1
Wheat bran	5
Limestone	1.32
Dicalcium phosphate	0.5
Salt	0.2
Sodium bicarbonate	0.1
Choline chloride	0.05
Calcium formate	0.15
Montmorillonite	0.1
Lysine (70%)	0.25
Methionine	0.08
Bacillus	0.05
Complex phosphoesterasum	0.02
Mineral premix	0.12
Vitamin premix	0.06
Total	100
Calculation of nutrients	
Metabolizable energy MJ/kg	12.12
Crude protein	14.30
Calcium	0.68
Methionine	0.31
Lysine	0.76
Total phosphorus	0.49

**Table 2:** The main components of DNL.

Name	Content (%)
Total polysaccharides	9.47
Alkaloids	0.41
Crude protein	5.01
Crude fiber	72.01
Metabolizable energy MJ/kg	8.91

### Semen collection and analysis

Semen was collected from the 20 roosters on Days 0, 20, 45, and 60 of the experiment using the massage semen collection method as previously described [17], and the amount of semen collected was recorded. Fresh semen (100 µ L) was diluted with pre-warmed saline at a ratio of 1:175. The number of sperm in five squares was counted using a blood cell counter [18] under an optical microscope at 400 × magnification. Each sample was counted in triplicates. Sperm density was calculated as follows:


$$Spermdensity\left(\frac{pcs}{mL}\right)=\left(\frac{5-squarespermcount}{80}\right)\times 400\times 10\times 175\times 1000$$

Sperm motility and density were determined using a similar method. Normal saline at 37 °C was used as the diluent, and the heating plate of the microscope was adjusted to 37 °C. The number of nonlinear motion sperms, such as those exhibiting immobility, *in situ* rotation, and rotation in five squares, was counted. The number of nonlinear motion sperms per milliliter of semen was subsequently calculated. Then, the temperature of the heating table was increased to 45 °C, and the heating table was left on for 5 min. Sperm were counted, the number of sperm in five squares was counted, and the total number of sperm per milliliter of semen was calculated. Sperm motility was calculated as follows:


$$Spermmotility=\frac{totalspermcount-nonlinearspermcount}{totalspermcount}$$

### Determination of egg hatching rates

Semen was collected on Day 45 of the experiment to determine the reproductive performance of roosters. Next, a batch of hens raised in the same environment was selected for artificial insemination. In each group, 150 eggs that met the hatching requirements for incubation were selected. The number of non-fertilized, fertilized, and dead embryonic eggs was recorded twice during the incubation period, and the fertilization rate was calculated. The number of chicks was recorded on Day 21 of incubation. The fertilization rates, as well as hatching rates of fertilized and hatched eggs were calculated as follows:


$$Fertilizationrate=\left(\frac{numberoffertilizedeggs}{numberofeggs}\right)\times 100$$

$$Hatchingrateoffertilizedeggs=\left(\frac{hatchlings}{fertilizedeggs}\right)\times 100$$

$$Hatchingrateofhatchedeggs=\left(\frac{hatchednumber}{hatchedeggnumber}\right)\times 100$$

### Sampling

Blood samples (4 mL) were collected from the wing veins of 20 fasting roosters on Days 0, 40, and 60 of the experiment. The serum was separated through centrifugation at 3000 r/min for 15 min at 4 °C and stored at -80 °C. At the end of the experiment, 20 roosters were weighed. Ten roosters from each group were euthanized using cervical dislocation, and the testes were immediately removed.

The weights of the left and right testes were measured using an electronic balance, and the testis index was calculated as follows:


$$Testicularindex=\left(\frac{totaltesticularweight}{bodyweight}\right)\times 100$$

Some of the collected testes were immediately fixed in 4% paraformaldehyde solution for histological analysis. The remaining testes were immediately stored at -80 °C for oxidative status, gene expression, and metabolomic analyses.

### Measurement of reproductive hormones

The serum concentrations of luteinizing hormone (LH), folliclestimulating hormone (FSH) and testosterone were measured by Enzyme Linked Immunosorbent Assay (ELISA) using a commercial ELISA kit for chicken (Nanjing Jiancheng Bioengineering Institute, China) according to the manufacturer’s instructions. All assays were performed in 96-wellplates and the absorbance was measured at 450 nm using a Microplate Reader (Synergy H4, Biotek Instruments, USA). A standard curve was drawn for the determination of hormone levels.

### Measurement of antioxidant indices

Serum samples were used to analyze antioxidant enzyme activities. Frozen testes (0.2 g) were homogenized in 2 mL cold saline using a mortar and pestle. The homogenate was centrifuged at 12000 × *g* at 4 °C for 10 min, and the supernatant was collected to evaluate the oxidation state. Antioxidant indicators included glutathione peroxidase (GSH-Px), total superoxide dismutase (T-SOD), catalase (CAT), and total antioxidant capacity (T-AOC). Furthermore, the lipid metabolite malondialdehyde (MDA) was analyzed using spectrophotometry. The assays were performed using commercial kits (Nanjing Jiancheng Institute of Bioengineering, Nanjing, China) as previously described [19].

### qRT-PCR analysis

The relative expression levels of Nrf2 pathway-related genes nuclear factor E2-related factor 2 (*Nfr2*), superoxide dismutase (*SOD1*), catalase (*CAT*), quinone oxidoreductase-1 (*NQO-1*), heme oxygenase-1 (*HO-1*), and glutathione s-transferase 3 (*GSTA3*), as well as testosterone synthesis-related gene 17β-hydroxysteroid dehydrogenase (*HSD17b3*) in testicular tissue were determined using real-time quantitative polymerase chain reaction (qRT-PCR). Total RNA was isolated from 0.1 g testicular samples using the TRIzol reagent (Invitrogen, Carlsbad, CA, USA). Next, the purity and integrity of the isolated RNA were evaluated using agarose gel electrophoresis. Total RNA concentration was measured using a spectrophotometer. The First cDNA Synthesis Kit (#K1621; Thermo Fisher Scientific, Waltham, MA, USA) was used to reverse transcribe total RNA into cDNA, and the StepOne Plus Real-time PCR system (Forster, California, USA) was used for quantitative PCR analysis. The PCR conditions were as follows: Pre-denaturation at 95 °C for 2 min; 95 °C denaturation for 15 s, annealing at 60 °C for 15 s, extension at 72 °C for 1 min, 40 cycles; and 72 °C for 5 min to generate a melting curve and subsequently verify the amplification specificity. The relative gene expression levels were determined using the 2^− ΔΔCt^ method, with β-actin as the internal reference. Primer sequences are listed in S1 Table.

### Testicular metabolomic analysis

Metabolomic analyses were performed on six testicular samples from each group. After the sample was slowly thawed at 4 °C, an appropriate amount of sample was added to a pre-cooled methanol/acetonitrile/water solution (2:2:1, v/v). The mixture was vortexed and mixed, sonicated at low temperatures for 30 min, stored at -20 °C for 10 min, and centrifuged at 14000 × *g* for 20 min at 4 °C. The supernatant was vacuum dried, and 100 μL acetonitrile aqueous solution (acetonitrile:water = 1:1, v/v) was added for reconstitution during mass spectrometry, vortexed, centrifuged at 14000 × *g* for 15 min at 4 °C, and the supernatant was used for further analysis.

Samples were separated using the 1290 Infinity LC ultra-high-performance liquid chromatography (UHPLC) HILIC column (Agilent Technologies, Santa Clara, CA, USA) as follows: column temperature, 25 °C; flow rate, 0.5 mL/min; and injection volume, 2 μL. The mobile phase comprised water + 25 mM ammonium acetate + 25 mM ammonia (A) and acetonitrile (B). The gradient elution procedure was as follows: 0 to 0.5 min, 95% B; 0.5 to 7 min, B linearly changed from 95% to 65%; 7 to 8 min, B linearly changed from 65% to 40%; 8–9 min, B remained at 40%; 9 to 9.1 min, B linearly changed from 40% to 95%; and 9.1 to 12 min, B remained at 95%. The samples were placed in an automatic sampler at 4 °C throughout the analyses. Furthermore, continuous analysis of samples was carried out in a random order to avoid the influence of instrument detection signal fluctuations. Quality control samples were inserted into the sample queue to monitor and evaluate the stability and reliability of the system. Primary and secondary spectra of the samples were collected using an AB Triple TOF 6600 mass spectrometer. The samples were separated using the Agilent 1290 Infinity UHPLC and analyzed using a Triple TOF 6600 mass spectrometer (AB SCIEX, Framingham, MA, USA). Electrospray ionization (ESI) positive and negative ion modes were used for detection. The parameters of the ESI source were as follows: nebulizer-assisted heating gas 1 (Gas1): 60, assisted heating gas 2 (Gas2): 60, curtain gas (CUR): 30 psi, ion source temperature: 600 °C, spray voltage (ISVF): ± 5500 V (positive and negative modes); first-order mass-to-charge ratio detection range: 60–1000 Da, second-order ion mass-to-charge ratio detection range: 25–1000 Da, first-order mass spectrometry scan accumulation time: 0.20 s/spectra, and second-order mass spectrometry scan accumulation time: 0.05 s/spectra. Secondary mass spectrometry data were obtained through data-dependent acquisition mode (IDA) at a dynamic exclusion of isotope ions range of 4 Da. The peak intensity screening mode was used, and each scan collected 10 fragments. Cluster voltage (DP): ± 60 V (positive and negative modes), collision energy: 35 ± 15 eV.

### Histomorphology of the testes

The collected left testis was cut into small pieces (1.0 cm long, 1.0 cm wide, 0.5 cm high) and fixed in 4% paraformaldehyde solution for 48 h. The samples were further embedded in paraffin blocks, cut into 5 μm thick sections, and stained with hematoxylin-eosin. Then, two sections were prepared from each testicular sample. Ten curved seminiferous tubules with regular contours were randomly selected, and the diameter of the seminiferous tubules was measured at 400 × magnification using Case Viewer 2.3 (Wuhan Sevier Biotechnology Co., Ltd., Wuhan, China).

### Statistical analyses

Statistical analyses were performed using SPSS software (version 25.0; IBM Corp., Armonk, NY, USA). The data were analyzed using a t-test, and *P* < 0.05 was considered statistically significant. The data are expressed as the mean ± SEM and plotted using GraphPad Prism version 9.0 (GraphPad Software, Boston, MA, USA).

UPLC-MS/MS analysis was performed. Predictive variable importance (VIP) > 1.0 and fold change (FC) > 1.5 or *P* < 0.5 were the criteria for screening differential metabolites. Orthogonal partial least squares discriminant analysis (OPLS-DA) was used to extract VIP values. Score and permutation plots were subsequently generated using the MetaboAnalystR package in R 4.12 (R Foundation for Statistical Computing, Vienna, Austria).

The relationships between semen quality traits, testicular antioxidant parameters, antioxidant gene mRNA expression, and differential metabolites were analyzed by calculating the Spearman correlation coefficient. Analysis and mapping by online software (https://www.omicstudio.cn/tool).

## Results

### Effects of dietary DNL supplementation on semen quality in roosters

**Fig 1:**
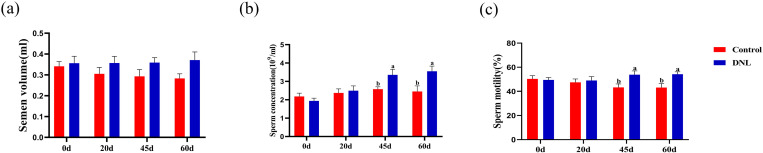
Effect of DNL supplementation on the semen quality of roosters. (a) semen volume. (b) sperm concentration. (c) sperm motility. The data represent mean ± SEM (n = 10). Different letters indicate significant differences (*P* < 0.05).

As shown in Fig 1, there was no significant difference in semen quality between the control and DNL groups on Day 0 of the experiment (*P* > 0.05). Daily supplementation of DNL did not affect seminal volume. The sperm density and motility in the DNL group were significantly higher than those in the control group (*P* < 0.05) on Days 45 and 60.

### Effects of dietary DNL on the hatching rate of eggs

At the same amount and depth of insemination. The fertility, hatchability of hatching eggs and hatchability of fertilized eggs in DNL group were 87.33%, 82.67% and 94.66%, respectively. The fertility, hatchability of hatching eggs, and hatchability of fertilized eggs in the control group were 80.00%, 71.33%, and 89.17%, respectively (Table 3).

**Table 3:** Effect of adding DNL on breeding performance of roosters.

Group	Fertility	Hatchability of hatching eggs	Hatchability of fertilized eggs
Control	80.00%	71.33%	89.17%
DNL	87.33%	82.67%	94.66%

### Effects of dietary DNL on serum hormone levels

**Fig 2:**
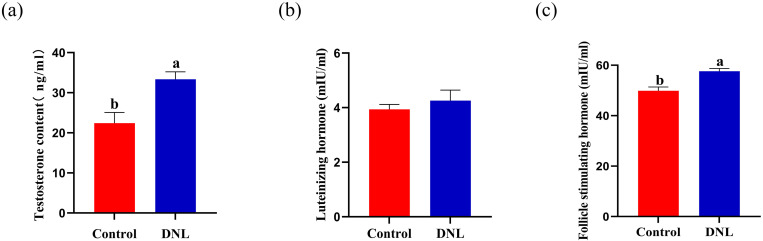
Effect of DNL supplementation on roosters’ serum hormone levels serum hormone levels. (a) Testosterone. (b) Luteinizing hormone (LH). (c) Folliclestimulating hormone (FSH). The data represent mean ± SEM (n = 8). Different letters indicate significant differences (*P* < 0.05).

On Day 60, the serum levels of testosterone and FSH in the DNL group were significantly higher than those in the control group (*P* < 0.05; Fig 2). In contrast, there was no significant difference in serum LH levels between the two groups (*P* > 0.05).

### Effects of dietary DNL on serum antioxidant indices

**Fig 3:**
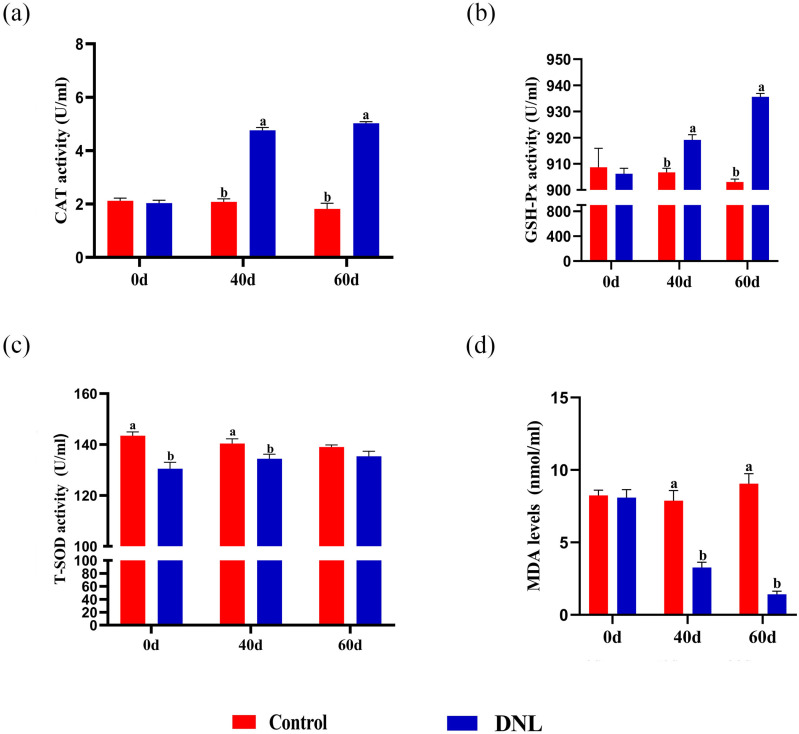
Effect of DNL supplementation on roosters’ serum antioxidant enzyme activity. (a) Catalase (CAT). (b) Glutathione peroxidase (GSH-Px). (c) Total superoxide dismutase (T-SOD). (d) Malondialdehyde (MDA). The data represent mean ± SEM (n = 8). Different letters indicate significant differences (*P* < 0.05).

There was no significant difference in serum CAT, GSH-Px activity, and MDA levels between the control and DNL groups on Day 0 of the experiment (*P* > 0.05). In addition, T-SOD activity was significantly higher than that in the control group (*P* < 0.05). CAT and GSH-Px activities in the DNL group were significantly higher than those in the control group on Days 40 and 60 (*P* < 0.05). Moreover, there was no significant difference in serum T-SOD activity between the DNL group and the control group on Days 60 (*P* > 0.05). The serum MDA level in the DNL group was significantly lower than those in the control group on Days 40 and 60 (*P* < 0.05; Fig 3).

### Effects of dietary DNL supplementation on the testicular tissue morphology of roosters

**Fig 4:**
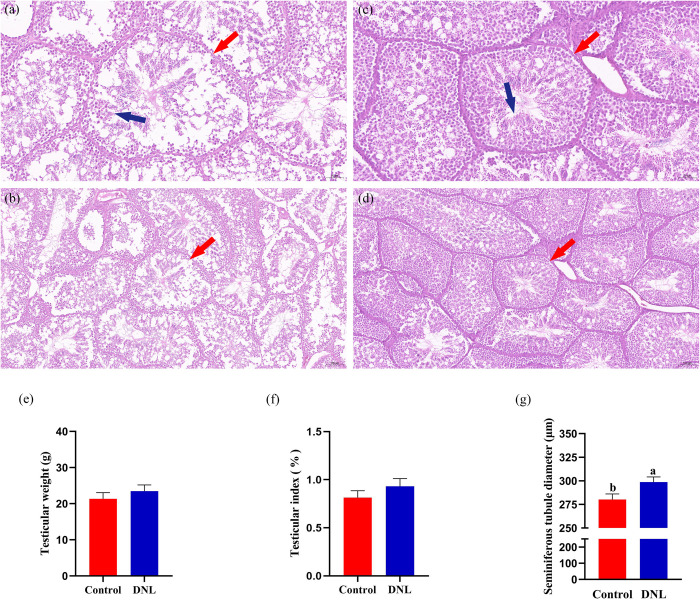
Effect of DNL supplementation on the Testicular morphology of roosters. (a) Control testicular tissue, Scale bar: 50 μm. (b) Control testicular tissue, Scale bar: 100 μm. (c) DNL testicular tissue, Scale bar: 50 μm. (d) DNL testicular tissue, Scale bar: 100 μm. Red arrow: spermatogenic tubules, Blue arrow: Sperm cells. (e) testicular weight. (f) testicular index. (g) seminiferous tubule diameter. The data represent mean ± SEM (n = 10). Different letters indicate significant differences (*P* < 0.05).

There was no significant difference in the testicular weight or index between the DNL and control groups (*P* > 0.05; Figs 4e and 4f). However, there were more supporting sperm cells in the seminiferous tubules, tight intercellular spaces, thicker seminiferous epithelium, and less vacuolization in the DNL group (Figs 4a-4d). Furthermore, the average diameter of the seminiferous tubules in the DNL group was significantly higher than that in the control group (*P* < 0.05; Fig 4g).

### Effects of dietary DNL supplementation on testicular antioxidant indices

**Fig 5:**
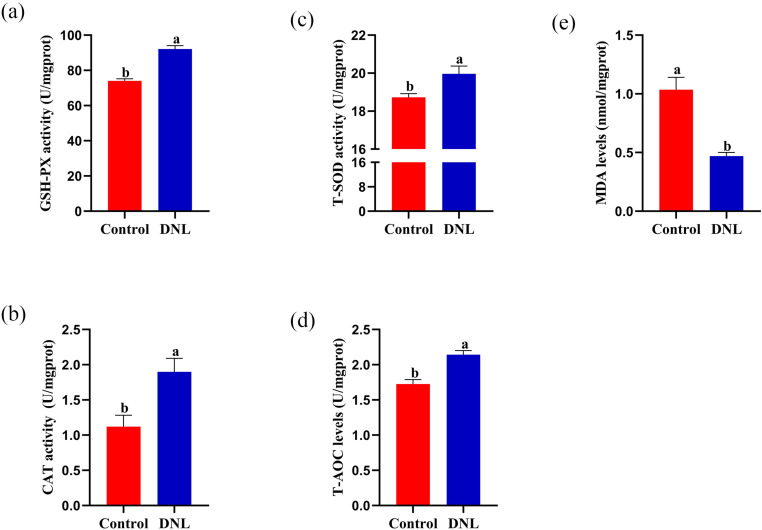
Effect of DNL supplementation on the testis antioxidant enzyme activity of roosters. (a) Glutathione peroxidase (GSH-Px). (b) Catalase (CAT). (c) Total superoxide dismutase (T-SOD). (d) Total antioxidant capacity (T-AOC). (e) Malondialdehyde (MDA). The data represent mean ± SEM (n = 10). Different letters indicate significant differences (*P* < 0.05).

GSH-Px, CAT, and T-SOD activities in the testicular tissue of the DNL group were significantly higher than those in the control group (*P* < 0.05; Fig 5). Similarly, the total antioxidant capacity (T-AOC) levels were significantly higher than those in the control group (*P* < 0.05). In contrast, the MDA levels were significantly lower than those in the control group (*P* < 0.05).

### Effects of the relative mRNA expression of genes in the testes

**Fig 6:**
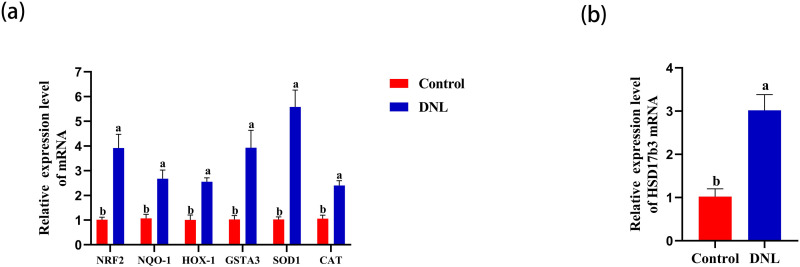
Effects of adding DNL on antioxidant related genes and HSD17b3 in rooster testis. (a)nuclear factor erythroid 2-related factor 2 (Nrf2), quinine oxidoreductase-1 (NQO-1), heme oxygenase-1 (HOX-1), glutathione S-transferase alpha 3 (GSTA3), superoxide dismutase 1 (SOD1), catalase (CAT). (b)hydroxysteroid 17-beta dehydrogenase 3 (HSD17b3). The data represent mean ± SEM (n = 6). Different letters indicate significant differences (*P* < 0.05).

The relative expression levels of *Nrf2*, *NQO-1*, *HOX-1*, and *GSTA3* in the DNL group were significantly higher than in the control group (*P* < 0.05). In addition, the relative expression levels of *SOD1* and *CAT* in the DNL group were significantly higher than in the control group (*P* < 0.05; Fig 6a). Similarly, the relative expression of *HSD17b3* in the DNL group was significantly higher than that in the control group (*P* < 0.05; Fig 6b).

### Correlation analysis between semen quality and antioxidant parameters

**Fig 7:**
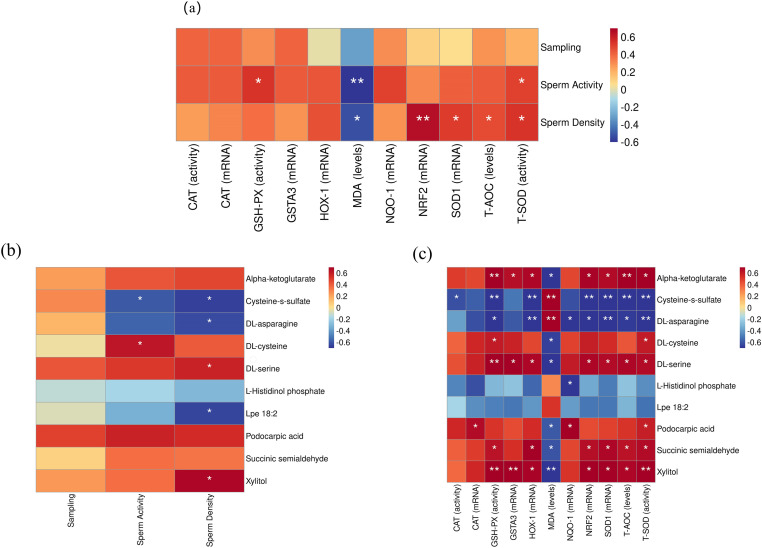
Spearman’s correlation analysis heatmap. (a)semen quality traits and testis antioxidant parameters. (b)differential metabolites and semen quality traits. (c)differential metabolites and testis antioxidant parameters. *p < 0.05, **p < 0.01.

As shown in Fig 7a, sperm density was significantly positively correlated with T-SOD activity, T-AOC levels, and *SOD1* expression (*P* < 0.05); extremely significantly positively correlated with *Nrf2* expression (*P* < 0.01); and significantly negatively correlated with MDA levels (*P* < 0.05). Moreover, sperm motility was positively correlated with T-SOD and GSH-Px activity (*P* < 0.05) and negatively correlated with MDA levels (*P* < 0.01).

### Testicular metabolomic analysis

**Fig 8:**
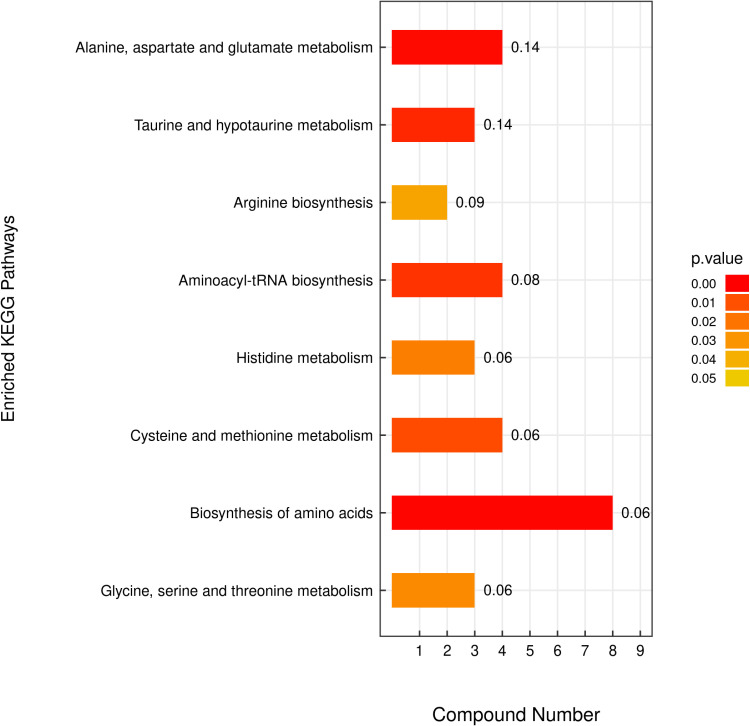
KEGG enrichment analysis of differential metabolites.

In total, 1553 metabolites were identified in the testes of the control and DNL groups. PCA and OPLS-DA analyses (S1 Fig) showed a significant separation between these two groups. The screening criteria of VIP > 1 and FC > 1.5 or *P* < 0.05 were used to screen the differential metabolites. As shown in S2 Table, 38 differential metabolites were screened in the anion model between the control and DNL groups. Of these, the expression of 18 was downregulated whereas that of 20 was upregulated. Similarly, 38 differential metabolites were identified in the cation model. Of these, the expression of 20 was downregulated and that of 18 was upregulated. KEGG pathway enrichment analysis (Fig 8) showed that most of the differential metabolites between the control and DNL groups were enriched in pathways such as amino acid biosynthesis; alanine, aspartic acid, and glutamic acid metabolism; taurine and hypotaurine metabolism; cysteine and methionine metabolism; histidine metabolism; as well as glycine, serine, and threonine metabolism.

Correlation analysis showed a positive correlation between sperm motility and cysteine (*P* < 0.05). In contrast, sperm motility was negatively correlated with cysteine-S-sulfate (*P* < 0.05). Sperm density was significantly positively correlated with serine and xylitol (*P* < 0.05) and significantly negatively correlated with cysteine-S-sulfate, Lpe 18:2, and asparagine (*P* < 0.05; Fig 7b). Serine, cysteine, α-ketoglutarate, and xylitol were significantly (*P* < 0.05) or extremely significantly (*P* < 0.01) positively correlated with most antioxidant-related parameters (Fig 7c). Additionally, they were significantly (*P* < 0.05) or extremely significantly (*P* < 0.01) negatively correlated with MDA levels. Cysteine-S-sulfate and asparagine were significantly (*P* < 0.05) or extremely significantly (*P* < 0.01) negatively correlated with most antioxidant-related parameters and extremely significantly positively correlated with MDA levels (*P* < 0.01).

## Discussion

Semen quality facilitates the reproductive performance of roosters because the fertilization rate of roosters is positively correlated with sperm motility, concentration, and motility [20]. In this study, we evaluated the effects of dietary DNL supplementation on the semen quality of aged roosters. Sperm density and motility in the DNL group significantly increased on Days 45 and 60. These results are consistent with previous studies [12–14], which revealed a significant improvement in semen quality indices of mice after treatment with *Dendrobium* extract. This indicates that dietary supplementation with DNL improved semen quality in aged roosters. Although the mechanism by which dietary DNL supplementation affects semen quality remains unclear, it may be related to antioxidant defense systems.

The mechanism through which age negatively affects semen quality and reproductive performance is related to oxidative stress [21]. The testes are sites of spermatogenesis, which is easily affected by oxidative stress [22]. ROS levels in tissues increase with aging [4]. OS caused by excessive ROS accumulation in the testes can lead to an abnormal testicular structure, loose arrangement of spermatogenic cells, and vacuolization of seminiferous tubules [23,24]. Histological changes such as degeneration and atrophy of seminiferous tubules occur in the testes of aging roosters. This includes thinning of the seminiferous epithelium and decreased diameter of the seminiferous tubules [25]. *Dendrobium* extract can improve the destruction of testicular tissue structures in mice and increase the number of spermatogenic cells [12,14]. In the present study, DNL supplementation delayed the degeneration of testicular tissue in aging roosters, as demonstrated by an increase in the diameter of seminiferous tubules, increased spermatogenic cells, and less vacuolization of seminiferous tubules. This result is consistent with previous studies. Compared with low sperm motility roosters, the structure of seminiferous tubules of high sperm motility roosters is more complete and the diameter of seminiferous tubules is larger [26].

Oxidative stress in testicular tissues can disrupt DNA, RNA, and protein functions in sperm and other testicular cells, resulting in cellular damage. The resulting defective sperm can lead to a decline in male reproductive performance [21]. Moreover, excessive ROS production reduces cell membrane fluidity, damages mitochondria, and reduces enzyme activity by causing a lipid oxidation reaction on the sperm cell membrane, thereby decreasing semen quality [27]. MDA is the main lipid peroxidation degradation product induced by ROS and reflects the level of oxidative stress [28]. Antioxidant enzymes such as SOD, CAT, and GSH-Px constitute a crucial antioxidant defense system [29] that is vital for maintaining male reproductive health [7]. In this study, serum CAT and GSH-Px activities significantly increased in the DNL group, whereas MDA levels significantly decreased. T-SOD, CAT, GSH-Px activities and T-AOC levels in the testes were significantly higher than those in the control group. Contrastingly, MDA levels were significantly lower than in the control group. Sperm density and motility were significantly negatively correlated with MDA levels and positively correlated with the activity of some antioxidant enzymes. As a natural antioxidant, *Dendrobium* improves the antioxidant capacity in the body [30]. Zhao et al. [16] found that dietary *Dendrobium* improved broiler growth performance, antioxidant capacity, and intestinal health. Xu et al. [31] found that adding *Dendrobium* polysaccharides to the diet of aging mice improved their antioxidant capacity and intestinal mucosal barrier integrity. In the present study, DNL dietary supplementation improved the antioxidant capacity of aged roosters. This was closely related to improved semen quality.

Antioxidant enzyme activities are closely related to gene expression [32]. The Nrf2 signaling pathway regulates the antioxidant defense system in animals. The activation of Nrf2 can upregulate the expression of downstream genes such as *SOD*, *GSH-Px*, *GST*, *CAT*, *HO-1*, and *NQO1* to alleviate oxidative stress [33]. The expression levels of *Nrf2*, *GST*, *HO-1*, *NQO1*, *SOD1*, and *CAT* in the DNL group were significantly higher than those in the control group and were positively correlated with semen quality. *Nrf2* expression was positively correlated with sperm density. Similar results were observed when the diet was supplemented with other antioxidant compounds in previous studies. Dietary supplementation with α-lipoic acid enhanced the antioxidant capacity of the testes through the Nrf2 signaling pathway, played an anti-apoptotic role, and improved semen quality in elderly roosters [28]. Gao et al. [9] found that natural astaxanthin may improve semen quality by increasing antioxidant enzyme activity and scavenging hydroxyl radicals, which may be related to upregulation of the MAPK/Nrf2 pathway. These results indicate that DNL enhances the antioxidant capacity of aging roosters by regulating the Nrf2 pathway to improve semen quality.

High ROS levels can reduce male reproductive performance by directly inducing oxidative stress and indirectly affecting the hypothalamic-pituitary-adrenal axis [34]. Excessive ROS production can reduce male sex hormone levels (including testosterone, FSH, and LH), disrupt the balance of hormones that regulate male reproductive function, and lead to a decline in reproductive performance [35]. FSH and LH secreted by the pituitary gland and testosterone secreted by Leydig cells regulate spermatogenesis [36]. The FSH receptor is located on the membrane of Sertoli cells, whereas the LH receptor is located on the membrane of interstitial cells. These receptors coordinate testosterone synthesis to maintain normal spermatogenesis, sperm health, and density [37]. In addition, testosterone secretion and synthesis are related to the steroidogenic enzyme 17b-hydroxysteroid dehydrogenase (HSD17) [2]. Testosterone is an essential hormone for spermatogenesis, particularly in older roosters. Thus, decreased testosterone secretion is a significant factor in the decrease in sperm concentration in aging roosters [25]. Ansari et al. [2] showed that d-aspartic acid could improve the semen quality of elderly roosters by increasing testosterone levels and altering gene expression. Dietary supplementation with linseed oil may improve semen quality by increasing testosterone secretion [8]. In this study, serum testosterone and FSH levels in the DNL group were significantly higher than those in the control group. In addition, the expression of *HSD17b3*, which is related to testosterone synthesis in testicular tissue, was significantly higher than that in the control group. Part of the increase in sperm concentration observed in this study may be related to increased reproductive hormones in the blood.

Metabolomics is a discipline that explores the dynamic changes in metabolites. Metabolites are endpoints of biochemical reactions that reflect the final effects of biological functions [38]. In this study, dietary supplementation with DNL changed the abundance of 76 metabolites in the testes of aged roosters, including lipids and lipid molecules, organic acids and their derivatives, and organic heterocyclic compounds. The 38 differential metabolites was significantly upregulated and 38 differential metabolites was downregulated compared to those in the control group. These differential metabolites were significantly enriched in pathways, such as amino acid biosynthesis, cysteine and methionine metabolism, as well as taurine and hypotaurine metabolism. The changes in DL-serine, DL-cysteine, and α-ketoglutarate levels may be related to improvements in the antioxidant capacity and reproductive performance of aged roosters.

DL-serine and DL-cysteine levels in the testes of the DNL group were significantly upregulated compared with those in the control group. DL-serine was significantly positively correlated with some antioxidant enzymes and genes and was significantly negatively correlated with MDA levels. DL-cysteine content was positively correlated with GSH-Px activity, negatively correlated with MDA levels, and positively correlated with sperm motility. Glutathione, an antioxidant, protects cellular components from ROS attacks [39]. Cysteine and glutamate are precursors of glutathione biosynthesis [40]. Serine, a cysteine precursor, increases GSH-Px activity by supporting glutathione synthesis and the methionine cycle [41]. It mainly synthesizes cysteine via condensation with homocysteine and provides a carbon unit for homocysteine re-methylation [42]. This process can alleviate oxidative stress by upregulating the expression of glutathione synthesis-related genes; increasing glutathione concentration [43]; reducing MDA concentrations; and increasing SOD, GSH-Px, and CAT concentrations [44]. Moreover, the addition of cysteine to the filler during semen storage can prevent the loss of sperm motility in roosters [45,46]. These findings suggest that changes in cysteine and serine levels in the testes enhance their antioxidant capacity through the biosynthesis of glutathione, which improves sperm motility in aged roosters. In addition, α-ketoglutarate contents in the DNL group were significantly upregulated compared with the control group in this study. Here, α-ketoglutarate was positively correlated with Nrf2, HOX-1, and GSTA3 expression and the activity of some antioxidant enzymes. In contrast, it was negatively correlated with MDA levels. As a glutamic acid and glutamine precursor and a central molecule in the tricarboxylic acid (TCA) cycle, α-ketoglutarate improves the energy metabolism and antioxidant capacity of the body [47]. Furthermore, α-ketoglutarate can increase SOD, GSHPx, and CAT activities; reduce MDA levels; and play an important role in ROS scavenging [48]. This antioxidant effect is related to the activation of the Nrf2 pathway [49].

## Conclusion

Our results indicate that dietary supplementation with DNL may improve the semen quality and testicular structure of aged roosters by enhancing antioxidant capacity, regulating the expression of related antioxidant genes, increasing reproductive hormone levels, and changing testicular metabolism. This subsequently leads to increased DL-serine, DL-cysteine, and α-ketoglutarate production. These findings indicate that DNL can be used as a feed additive to improve the reproductive performance of aged roosters.

## Supporting information

S1 Fig PCA analysis and OPLS-DA analysis between control and DEL groups. (a)PCA analysis negative model (b)PCA analysis positive model (c)OPLS-DA analysis negative model (d)OPLS-DA analysis positive model.
(TIF)

S1 Table Primer sequences for qRT-PCR. (DOCX)

S2 Table Screening of testis differential metabolites. (XLSX)

## List of abbreviations

DNL: *Dendrobium nobile* Lindl
ROS: Reactive oxygen species
OS: oxidative stress
GSH-Px: glutathione peroxidase
T-SOD: total superoxide dismutase
CAT: catalase
T-AOC: total antioxidant capacity
FSH: follicle-stimulating hormones
LH: luteinizing hormones
MDA: malondialdehyde
*Nfr*2: nuclear factor E2-related factor 2
*SOD1*: superoxide dismutase
*NQO-1*: quinone oxidoreductase-1
*HO-1*: heme oxygenase-1
*GSTA3*: glutathione-S-transferase 3
*HSD17b3*: 17β-hydroxysteroid dehydrogenase
qRT-PCR: real-time quantitative polymerase chain reaction
ESI: electrospray ionization
UHPLC: LC ultra-high-performance liquid chromatography
VIP: predictive variable importance
FC: fold change
SEM: standard error of mean
OPLS-DA: orthogonal partial least squares discriminant analysis
