# Supplementary figures and images for: Effects of Dendrobium nobile on antioxidant capacity, hormone levels, testicular metabolism, and reproductive performance of aged roosters
Source: PLoS One. 2025 May 9;20(5):e0322853. doi: 10.1371/journal.pone.0322853 (PMC12064193; doi:10.1371/journal.pone.0322853)

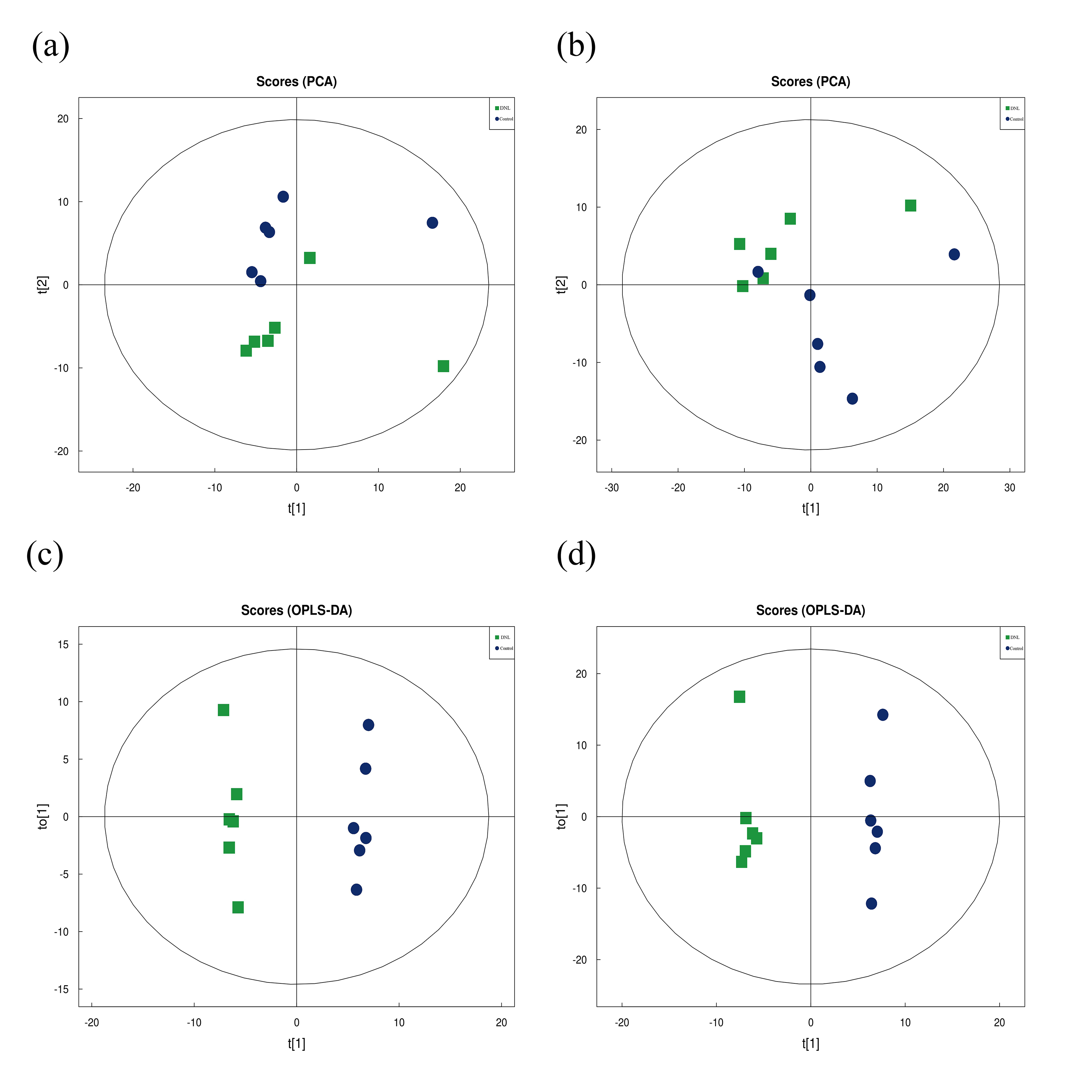

Supplement: S1 Fig — (a)PCA analysis negative model (b)PCA analysis positive model (c)OPLS-DA analysis negative model (d)OPLS-DA analysis positive model. (TIF) [file pone.0322853.s001.tif]
