# Supplementary material for: Effects of Dendrobium nobile on antioxidant capacity, hormone levels, testicular metabolism, and reproductive performance of aged roosters
Source: PLoS One. 2025 May 9;20(5):e0322853. doi: 10.1371/journal.pone.0322853 (PMC12064193; doi:10.1371/journal.pone.0322853)
Supplement: S1 Table — (DOCX) [file pone.0322853.s002.docx]

**S1 Table** **Primer sequences for qRT-PCR**

| Gene | Primer sequences |
| --- | --- |
| *Nrf2* | Forward: CTGTCTTCAGCCTTTTTGGACA |
|  | Reverse: AAAAACTTCACGCCTTGCCC |
| *HO-1* | Forward: CACACAACGCTGAAAGCATGTC |
|  | Reverse: AACTCGTGGAGGGACACCT |
| *NQO-1* | Forward: AACCTCTTTCAACCACGCCA |
|  | Reverse: TTCTTGAGGGGTCCGGTGAT |
| *GSTA3* | Forward: CGTCGTCCAACCAGCAGATA |
|  | Reverse: CCGTGGTCCTTCAAAACCTTC |
| *SOD1* | Forward: TGACCTCGGCAATGTGACTG |
|  | Reverse: CACTTTTTGCATGGACCACCA |
| *CAT* | Forward: GCTGAAGCTGGGAAAAAGGATG |
|  | Reverse: TCCTGCAGTTGTATGGACGC |
| *HSD17b3* | Forward: GAGAATGGGCAGTGGTCACA |
|  | Reverse: TATTTAGCCCGCGTTTTGCC |
| *β-actin* | Forward: GAGAAATTGTGCGTGACATCA |
|  | Reverse: CCTGAACCTCTCATTGCCA |
